# Editorial: Branching Morphogenesis During Embryonic Lung Development

**DOI:** 10.3389/fcell.2021.728954

**Published:** 2021-07-12

**Authors:** Elie El Agha, Dagmar Iber, David Warburton

**Affiliations:** ^1^Department of Internal Medicine, Universities of Giessen and Marburg Lung Center (UGMLC), Institute for Lung Health (ILH), Cardio-Pulmonary Institute (CPI), Member of the German Center for Lung Research (DZL), Justus-Liebig University Giessen, Giessen, Germany; ^2^Swiss Institute of Bioinformatics (SIB), ETH Zürich, Basel, Switzerland; ^3^Developmental Biology and Regenerative Medicine Program, Saban Research Institute, Children's Hospital Los Angeles, Los Angeles, CA, United States

**Keywords:** lung, branching morphogenesis, organogenesis, alveologenesis, organoids, FGF10

The process of branching morphogenesis has fascinated both experimental and theoretical biologists for decades. This developmental phenomenon is an integral part of organogenesis of the lung, kidneys, mammary glands, and salivary glands, to name a few. Importantly, the branching process is widely regarded as a rate-limiting morphogenic step during organogenesis, as it helps define organ size, shape, and architecture, thus allowing such organs to assume their final mature structure and achieve optimal functionality. During embryonic lung development, airway epithelial branching occurs concomitantly with proximal-distal patterning and gives rise to the respiratory tree, thus setting the stage for alveologenesis. The latter is a process whereby primitive alveolar sacs located at the terminus of a given airway are subdivided into the smallest respiratory units of the lung, the alveoli. This developmental stage starts *in utero* in humans while it takes place entirely postnatally in mice. Branching morphogenesis and subsequent alveolarization are important for achieving a significantly large surface area for efficient gas exchange in the fully developed lung.

In the “Branching Morphogenesis During Embryonic Lung Development” Research Topic of *Frontiers in Cell and Developmental Biology*, the state of the art, emerging concepts and approaches, open questions, and future directions related to this field of study are presented. Jones et al. provide a comprehensive overview regarding the cellular, molecular, and physical factors that control branching of the embryonic mouse lung, and how they relate to FGF10 signaling. Originally identified in *Drosophila melanogaster* as *branchless* and *breathless*, respectively (Klämbt et al., 1992; Sutherland et al., 1996), FGF10 and its cognate receptor FGFR2b have emerged as a key regulator of lung branching morphogenesis. FGF10 impacts lung branching through diverse mechanisms including, but not limited to, chemoattraction, cellular rearrangement, control of the mitotic spindle orientation, and ECM protein deposition (Bellusci et al., 1997; Warburton et al., 2010; El Agha and Bellusci, 2014; Herriges and Morrisey, 2014). The spatiotemporal expression pattern of this ligand-receptor pair is well-documented in the developing lung, and both, signaling pathways and physical forces, such as intraluminal pressure and mechanical stretching, have been shown to modulate its expression and activity. How such diverse mechanisms are coordinated and how they converge to produce a reproducible branching pattern in the lung remain an area of active research.

Mathematical modeling has emerged as a powerful approach to explain branching stereotypy not only in the lung but also in other branched organs such as the kidney. Lang et al. review mechanical and signaling models for the control of branching morphogenesis, and argue that a ligand-receptor-based Turing mechanism based on mesenchyme-derived morphogens (in particular FGF10 and GDNF) and epithelial receptors (FGFR2b and RET, respectively) likely enables stereotypic branching not only in the developing mouse lung, but also in the kidney (Menshykau et al., 2014, 2019). Focusing on the key characteristics of the branching process, they provide a detailed comparison between the branching program of these two organs. While the biased elongation of the epithelial tubes can be explained with fluid flow-induced shear stress, the determinants of the branching angles, and the details of the budding process remain to be determined. Once determined, a “universal” morphogenic program may become apparent, that highlights the principles of organ development, and offers new perspectives on repair/remodeling after injury.

Branching of the epithelial tree proceeds in parallel to the development of the vascular system. The alignment of these two branched networks raises a debate on whether one of the two systems guides the branching of the other. Kina et al. compile evidence that is in favor of each scenario and highlights the gaps that still need to be addressed in this context. The influence of the vascular system on epithelial branching has become ever more apparent, but better cell type-specific *in vivo* intervention strategies in addition to novel *ex vivo* approaches are still needed to precisely dissect the crosstalk between the airways and vessels during lung branching morphogenesis. In a related context, lung organoids have emerged as a powerful tool to model lung development and repair. Vazquez-Armendariz and Herold provide an overview of the various types of human and murine lung organoids that have been described so far and how they can be used to study lung branching. The challenge remains (1) achieving maximal cellular diversity that mirrors, or at least closely resembles, the native cellular *in vivo* composition and (2) minimizing the artifacts related to the culture conditions and affecting morphogenesis and/or response to injury.

A perspective on the potential involvement of conserved mechanisms in the formation of airways and alveoli is provided by Warburton. The idea stems from the observation that both domain branching of (proximal) embryonic airways and secondary septation of (distal) primitive alveolar sacs proceed *via* epithelial extrusion through an orifice. The orientation and stiffness of the orifice boundary would thus define the patterning process in both lung compartments. It is important to mention that 3D visualization of the alveolar network is more challenging than that of the “smaller” embryonic branched airway. Until recently, alveolar walls have been perceived as protruding finger-like structures due to the artifact of 2D imaging of thin lung sections. This conception has been challenged with the advent of 3D imaging of thick lung sections (Branchfield et al., 2016). Here, it is suggested that alveolar mouths are round purse string-like structures rather than fingers or ridges. If the resemblance between airway branching and alveolar septation is indeed proven to be true, this might have an impact on our understanding of alveologenesis, as our knowledge of airway morphogenesis has been facilitated by the simpler structure and ease of whole-mount imaging and organotypic culture. The gain of knowledge regarding developmental alveologenesis might help develop approaches to ultimately recapitulate this process for lung regeneration.

Finally, it is important to mention that the mechanisms controlling lung morphogenesis in mice are increasingly being validated in human fetal lung specimens although there are some key differences (Warburton, 2017; Nikolić et al., 2018; Danopoulos et al., 2019). Understanding developmental mechanisms controlling branching of the developing human lung might prove to be critical for managing many congenital/neonatal respiratory complications such as congenital diaphragmatic hernia, bronchopulmonary dysplasia, tracheoesophageal fistula, and other anomalies.

## Author Contributions

All authors contributed to drafting and editing the manuscript.

## Conflict of Interest

The authors declare that the research was conducted in the absence of any commercial or financial relationships that could be construed as a potential conflict of interest.
